# A case of methicillin‐resistant *Staphylococcus aureus* wound infection: phylogenetic analysis to establish if nosocomial or community acquired

**DOI:** 10.1002/ccr3.1442

**Published:** 2018-03-13

**Authors:** Francesco Cancilleri, Massimo Ciccozzi, Marta Fogolari, Eleonora Cella, Lucia De Florio, Alessandra Berton, Giuseppe Salvatore, Giordano Dicuonzo, Silvia Spoto, Vincenzo Denaro, Silvia Angeletti

**Affiliations:** ^1^ Department of Orthopaedic and Trauma Surgery University Campus Bio‐Medico of Rome Via Alvaro del Portillo 200 00128 Rome Italy; ^2^ Unit of Clinical Laboratory Science University Campus Bio‐Medico of Rome Via Alvaro del Portillo 200 00128 Rome Italy; ^3^ Infection control Committee University Campus Bio‐Medico of Rome Via Alvaro del Portillo 200 00128 Rome Italy; ^4^ Internal Medicine Department University Campus Bio‐Medico of Rome Italy

**Keywords:** MRSA infection, nosocomial infection control, phylogenetic analysis

## Abstract

Methicillin‐resistant *Staphylococcus aureus* (MRSA) infection is rapidly increasing in both hospital and community settings. A 71‐year‐old man admitted at the Department of Orthopaedics and Trauma Surgery, University Campus Bio‐Medico of Rome, with MRSA wound infection consequent to orthopedic surgery was studied and the MRSA transmission evaluated by phylogenetic analysis.

## Introduction

*Staphylococcus aureus* represents the most important cause of skin and soft tissue infections, osteomyelitis, and septic arthritis 1, 2, 3. Methicillin‐resistant *Staphylococcus aureus* (MRSA) infection is rapidly increasing in both hospital and community settings 4. Consequently, to the increasing prevalence of MRSA as so as to the decreased vancomycin susceptibility, complicating the infection treatment 5, a strict surveillance of the MRSA strains to limit their circulation and spread is required.

## Case Report

A 71‐year‐old man admitted at the Department of Orthopaedics and Trauma Surgery, University Campus Bio‐Medico of Rome, Italy, underwent to primary right hip arthroplasty by femur‐first surgical technique. Patient received antibiotic and venous thromboembolism prophylaxis with cefazolin and low molecular weight heparin, respectively. At discharge, none sign of local inflammation or infection was evident. After 20 days from discharge, patient was admitted at the Department of Orthopaedics and Trauma Surgery for the presence of secretion of the wound. A surgical toilet was performed, samples for wound bacterial culture were collected, and the patient was empirically treated with amikacin (1 g/die) and teicoplanin (200 mg × 2/die). Elevated serum C reactive protein (PCR) 131 mg/L (normal value, <3 mg/L was found. Multiple bacterial cultures were incubated on adequate culture media, colonies identified using MALDI‐TOF (MALDI Biotyper 3.0 software version – Bruker Daltonics, GmbH, Bremen, Germany) 6, and antimicrobial susceptibility test (AST) by Vitek2 Compact (bioMérieux, Marcy l'Etoile, France) performed in accordance with the EUCAST and the Clinical and Laboratory Standards Institute (CLSI) 7. Bacterial cultures resulted all positive for *Staphylococcus aureus*. The AST showed MIC values >4 mg/L for oxacillin, <0.5 mg/L for vancomycin, <0.5 g/L for teicoplanin, >8 mg/L for erythromycin, 2 mg/L for linezolid, 0.25 mg/L for clindamycin, <8 mg/L for amikacin, <0.03 mg/L for rifampicin, and <1 for tetracycline and doxycycline. Based on the MIC values, the strain resulted methicillin resistant (MRSA). Accordingly, the patient was treated with vancomycin (1 gr 2 × die) and amikacin (1 g/die). The MRSA phenotype was further confirmed using molecular test (Xpert MRSA assay, Cepheid) able to identify correctly *sccmecA* and *mecC* gene and to avoid false positive results due to empty cassettes.

During the treatment, a progressive decrease of the renal function was evidenced and antibiotic therapy was modified by replacing amikacin and vancomycin with rifampicin 450 mg × 2/die and linezolid (600 m × 2/die) with progressive improvement of the renal function. At discharge, patient was in good health status, the surgical wound appeared dyschromic without signs of dehiscence or fistulation and no pain (0 of 10) was reported in the Numerical rating scale (NRS). The prescription of the following antibiotic therapy (doxycycline 100 mg × 2/die) and rifampicin (450 mg × 2/die) for 45 days was performed.

After discharge, the Hospital Infection Control Committee aimed to analyze the route through which the patient could have acquired the *Staphylococcus aureus* MRSA infection of the wound applying phylogenetic analysis. A dataset containing *Staphylococcus aureus* MRSA *tuf* sequence from the patient plus seven *tuf* sequences from *Staphylococcus aureus* MRSA strains isolated at the University Hospital Campus Bio‐Medico of Rome and four from outpatients attending another hospital in Rome was built and used for the identification of *Staphylococcus* spp. at species and genus level as well as for phylogenetic analysis 8. Sequences were aligned using MAFFT online multiple sequence alignment program (https://mafft.cbrc.jp/alignment/server/). A transitions/transversions vs. divergence graph as well as the Xia's test of substitution saturation were implemented in DAMBE 9.

The nonparametric maximum parsimony model was chosen as best‐fitting model to minimize the number of events needed to explain the data. Statistical support for internal branches by bootstrap test (1000 replicates) was evaluated. The tree was built using MEGA 7 10 and visualized in FigTree 1.4.3. (http://tree.bio.ed.ac.uk/software/figtree).

**Figure 1:**
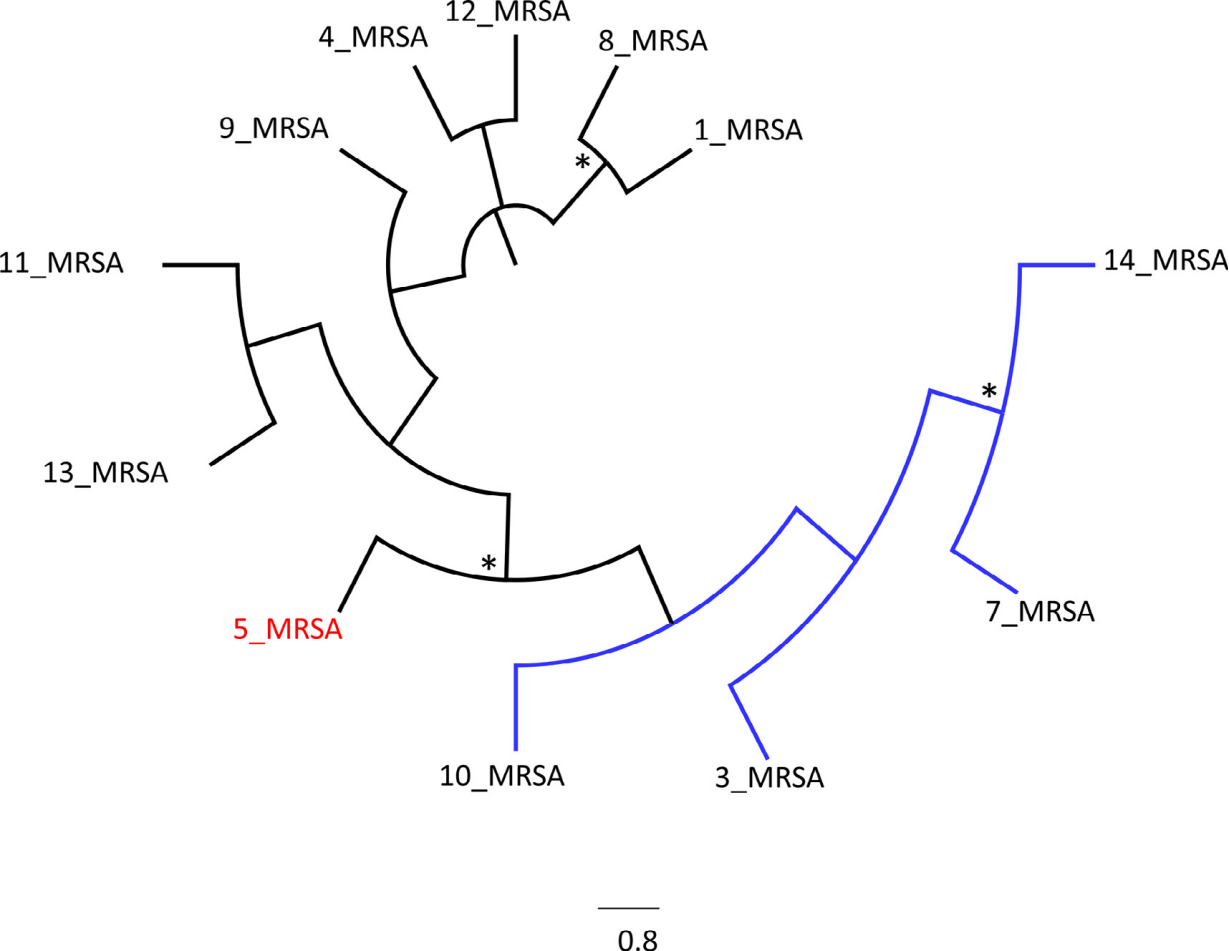
Maximum parsimony tree representing relatedness of DNA *tuf* gene sequences. *Symbol corresponds to bootstrap value more than 75%. Isolate from patient is marked in red.

The phylogenetic tree showed that the *tuf* sequence from *S.aureus* MRSA strain isolated in the patient (strain number 5) is positioned out of the cluster A including sequences from strains isolated in the University Hospital Campus Bio‐Medico (strains number 10, 3, and 7) and from another hospital in Rome (strain number 14) (Fig. 1). The phylogenetic analysis suggested that this strain was not related to other strains of cluster A (10, 3, and 7) circulating in the hospital setting where patient had been admitted, allowing to exclude the acquisition of the infection during the hospital stay. Otherwise, in Figure 1 is evident as strain number 5 from the patient could be considered as a sort of outgroup for the cluster A, indicating that strains 10, 3, and 7 probably came from strain number 5.

## Discussion

During last years, the circulation of MRSA strains has been reported, worldwide. Community‐acquired MRSA represents a specific clinical concern usually distinct from the hospital‐acquired strains, being typically isolated in patients without comorbidities and leaving far from healthcare system facilities. Community‐acquired strains are usually susceptible to clindamycin, tetracycline, and fluoroquinolones than hospital‐acquired strains that results mainly multi‐drug resistant 11.

Inert surfaces and apparatus contamination play a major role in cross‐transmission of pathogens and are responsible for nosocomial infection acquisition 12, 13.

Recently, Azarian et al. 14 used phylogenetic analysis to evaluate MRSA transmission between community and hospital settings and concluded that recurrent introductions within the intensive care unit represent a fundamental way for MRSA colonization maintenance.

In a recent longitudinal genomic surveillance, Coll et al. 15 demonstrated that in some cases, MRSA strains clustering together were isolated from patients having both community and hospital contact. Authors suggested that a strict surveillance of these strains has to be planned or reviewed.

In this case report, the phylogenetic analysis was used to define the possible route of acquisition of the MRSA wound infection. The aim was to understand if the patient has been infected by a nosocomial strain during his stay in the hospital or by a community strain later at his return back home. By this knowledge, a better nosocomial infection control could have been improved. The phylogenetic analysis applied to the MRSA strain isolated in our patient showed that the strain was probably acquired in the community setting being an outgroup for the cluster A including nosocomial strains.

In conclusion, the use of phylogenetic analysis, filling the gap of the clinical epidemiological investigation through the description of the transmission dynamic, represents an example of best practice surveillance in nosocomial setting.

## Authorship

FC, MC, SA, and VD: wrote and elaborated the manuscript. LDF, GD, and AS: performed the laboratory testing for the diagnosis. MC, EC, and MF: performed the phylogenetic analysis. FC, AB, and VD: performed the clinical diagnosis and the patients follow‐up. FC, MC, SA, SP and GD: evaluated the epidemiological and phylogenetic data. All authors: contributed to data analysis, drafting and revision of the manuscript and agree to be responsible for any aspect of the manuscript.

## Conflict of Interest

The authors declare that there is no conflict of interest regarding the publication of this manuscript.
